# Adverse effects of physiotherapy using the passive bicycle in the ICU

**DOI:** 10.1186/cc10199

**Published:** 2011-06-22

**Authors:** MM Martins, RY Sasai, MS Fole, B Rocha, EE Aquim, M Maturana

**Affiliations:** 1 Instituto Inspirar, Curitiba - PR, Brazil

## Introduction

The present study aimed to analyze the adverse effects of the therapy using the passive bicycle in the intensive care unit (ICU).

## Methods

This was a longitudinal, experimental, non-randomized controlled trial study. Performed with patients hospitalized in the ICU from Vita Curitiba and Batel Hospitals, and the Institute of Neurology from Curitiba, between 10 March and 30 June 2010. The total sample was 41 patients, with a total of 215 events, of both genders, being 23 men and 18 women, with an average age of 64 years, Glasgow average 11 ± 3 and APACHE II average score was 19 ± 6. Of the total sample, only two patients were evaluated according to the Ramsay scale, with an average of 4 ± 0.7. The passive bicycle activity was performed while the patient was in a bed or chair. The hemodynamic variables (heart rate, respiratory rate, mean arterial pressure and oxygen saturation) were collected at the beginning (before start of activity), 3 minutes after the start, and at the very end of the activity, and there was no pre-established activity time. The adverse effects accidental extubation; monitoring loss, like electrode, pulse oximetry and non-invasive blood pressure measures; change of balance, as lack of trunk control; fall; probe removal (nasogastric, nasoenteral and/or bladder); peripheral venous/arterial access were observed during the whole therapy time. The passive bicycle activity was performed 113 times in a chair (53%), and 102 times in bed (47%), having an average of 7.8 ± 2.29 minutes.

## Results

**Figure 1:**
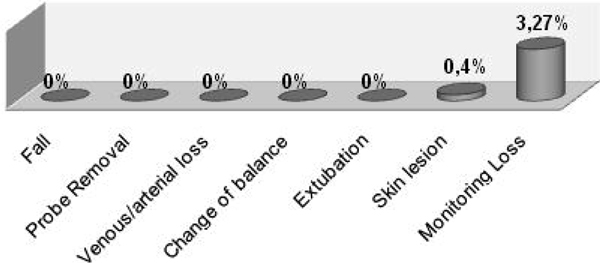
**Adverse effects during the passive bicycle activity**.

For the 215 events, were observed seven monitoring loss (3.27%) and one for skin lesion (0.467%), and there was no statistic significant from the proportion test. The adverse effects fall, probe removal, change of balance and extubation did not occur during the activity application. For the hemodynamic variables, using the Student *t *test (*P *< 0.05), mean arterial pressure, heart rate and respiratory rate, did not have significant change, without any hemodynamic instability during the activity (see Figure 1).

## Conclusion

The results show that using the passive bicycle in the ICU as a physiotherapy feature is secure and has a low risk of adverse effects related to ICU conduct.

